# Overexpression of Spexin 1 in the Dorsal Habenula Reduces Anxiety in Zebrafish

**DOI:** 10.3389/fncir.2019.00053

**Published:** 2019-08-14

**Authors:** Inyoung Jeong, Eunmi Kim, Jae Young Seong, Hae-Chul Park

**Affiliations:** ^1^Department of Biomedical Sciences, College of Medicine, Korea University, Ansan, South Korea; ^2^Department of Biomedical Sciences, Korea University, Seoul, South Korea

**Keywords:** zebrafish, anxiety, spexin 1, dorsal habenula, serotonergic system

## Abstract

Spexin (SPX) is an evolutionarily conserved neuropeptide that is expressed in the mammalian brain and peripheral tissue. Two orthologs are present in the teleost, SPX1 and SPX2. SPX1 is involved in reproduction and food intake. Recently, SPX1 neurons have been found to be located in the specific nuclei of dorsal habenula (dHb) and to project into the interpeduncular nucleus (IPN), in which galanin receptor 2a/2b (GALR2a/2b) expression was also observed. This indicates that habenula SPX1 neurons may interact with GALR2a/2b in the IPN; however, the function of SPX1 in the dHb-IPN neuronal circuit remains unknown. To determine the role of SPX1 in the dHb-IPN neural circuit, we generated transgenic zebrafish overexpressing SPX1 specifically in the dHb. We found that transgenic zebrafish overexpressing SPX1 in the dHb had anxiolytic behaviors compared with their wildtype siblings. Furthermore, quantitative PCR revealed that mRNA expression of *galr2a* and *galr2b* in the IPN and serotonin-related genes in the raphe was upregulated in the brains of transgenic zebrafish. Taken together, our data suggest that SPX1 function in the dHb-IPN neural circuits is implicated in the regulation of anxiety behaviors *via* modulation of the serotoninergic system in zebrafish.

## Introduction

Spexin (also known as SPX, NPQ, and C12ORF39) is a secreted neuropeptide that is mainly expressed in the brain, pancreas, kidney, liver, ovary, and adrenal glands of mammals and fish (Mirabeau et al., 2007; Sonmez et al., 2009; Porzionato et al., 2010; Rucinski et al., 2010; Liu et al., 2013; Wong et al., 2013). The functional mature peptide domain of SPX consists of 14 amino acids (NWTPQAMLYLKG^A^/_T_Q), which have multiple physiological functions, including adrenocortical cell proliferation, cardiovascular function, urine flow, nociception, reproduction, and food intake (Rucinski et al., 2010; Toll et al., 2012; Liu et al., 2013; Wong et al., 2013; Ma et al., 2018). Zebrafish have two SPX orthologs, SPX1 and SPX2, because of two-round whole-genome duplication (Kim et al., 2014). The amino acid sequence of zebrafish SPX1 differs from that of mammalian SPX by only one amino acid at the mature peptide domain (A↔T), suggesting that SPX functions might be evolutionarily conserved in zebrafish (Liu et al., 2013).

The habenula is a pair of small nuclei, which is involved in pain, stress, fear/anxiety, sleep, and reward behaviors in the brain (Hikosaka, 2010). The habenula consists of the medial habenula (MHb) and lateral habenula (LHb) in mammals. The MHb is a component of the evolutionarily conserved dorsal diencephalic conduction system, which is essential for anxiety and mood regulation (Yamaguchi et al., 2013; Hsu et al., 2016). The dorsal habenula (dHb) in zebrafish is the ortholog of the MHb in mice (Amo et al., 2010) and dHb-interpeduncular nucleus (IPN) circuitry is also involved in fear and anxiety responses (Agetsuma et al., 2010; Mathuru and Jesuthasan, 2013). We recently demonstrated that SPX1 neurons are located in the specific nuclei of dHb, which regulates fear and anxiety, and dHb SPX1 neurons project their axons to the galanin receptor 2a/2b (GALR2a/2b) neurons in the IPN of the zebrafish brain (Pandey et al., 2018; Kim et al., 2019). However, there is no direct evidence that dHb SPX1 function is linked to anxiety.

To assess the function of SPX1 in the dHb, we first generated *Tg(gpr151:gal4vp16);Tg(5xuas:spx1:p2a-mCherry)* zebrafish, which overexpressed SPX1 in the dHb, using the GAL4XUAS transactivator system. Subsequently, we analyzed the anxiety response using the novel tank test and expression of galr2a/2b and serotonin-related genes using quantitative RT-PCR in *Tg(gpr151:gal4vp16);Tg(5xuas:spx1:p2a-mCherry)* zebrafish.

## Materials and Methods

### Zebrafish Lines

All experimental procedures were approved by the Korea University Institutional Animal Care & Use Committee (IACUC) and performed in accordance with Animal experiment guidelines of Korea National Veterinary Research and Quarantine Service. *Tg(gpr151:gal4vp16*; Chou et al., 2016) and *Tg(5xuas:spx1:p2a-mCherry)* zebrafish were used in this study. All zebrafish were 6–8 month-old male adults, raised in a 14:10 light:dark cycle at 28.5°C.

### Plasmid Construct and Generation of Transgenic Zebrafish

To generate *Tg(5xuas:spx1:p2a-mCherry)* zebrafish, we performed PCR to amplify the spx1 ORF from cDNA using the following forward and reverse primers containing attB1 and attB2 sites: *spx1* forward, 5′-GGGGACAAGTTTGTACAAAAAAGCAGGCTCCATGA AAGATTTAAGGACTCTTGCGGCG-3′; *spx1* reverse, 5′-GGGGACCACTTTGTACAAGA AAGCTGGGTCCCAGTACGGCTCGTCTTCATCTCTG-3′. The PCR product containing attB sites was cloned into a middle entry vector using the BP reaction of the gateway system (Invitrogen). In addition, we cloned the 5′-entry clone containing five tandem repeated *uas* (5x*uas*) sequences and the 3′-entry clone containing the mCherry fused viral 2A peptide (Kim et al., 2011). The multi-site gateway LR reaction was performed using LR II clonase with entry clones, according to the manufacturer’s instruction (Invitrogen). To produce a stable transgenic zebrafish line, we injected the *5xuas:spx1:p2a-mCherry* DNA into one-cell embryos. To screen for germ-line transmitted transgenic zebrafish, we mated the *5xuas:spx1:p2a-mCherry* DNA-injected founder zebrafish with transgenic zebrafish containing gal4 and screened the progeny for mCherry expression. The obtained transgenic founder fish were crossed with wild-type zebrafish, and F1 transgenic zebrafish were raised to adulthood.

### Adult Brain Preparation

For the histological assay, 6–8-month-old male adult zebrafish (*n* = 5) were anesthetized using 200 mg/L of ethyl 3-aminobenzoate methanesulfonate salt (MS222, Sigma), until cessation of movement. Trans-cardiac perfusions were performed using phosphate buffered saline (PBS). Subsequently, the fish’s heads were decapitated and their brains were fixed in their entirety, using 4% paraformaldehyde. For total RNA preparation, 6–8-month-old adult zebrafish were sacrificed using the abovementioned procedures, with the exception of trans-cardiac perfusion and fixation.

### Whole-Mount *in situ* RNA Hybridization and Immunohistochemistry

To detect *spx1* and *gpr151* RNA transcripts, we used the antisense RNA probe against *spx1* and *gpr151*. The spx1 and gpr151-cloned vectors were linearized by restriction endonucleases (New England Biolabs) and the linearized vectors were transcribed using the dig-oxygenin labeling mixture (Roche). Whole-mount *in situ* RNA hybridization of the whole adult brains was performed as described previously (Thisse and Thisse, 2008). Subsequently, the whole-mounted RNA *in situ* hybridized brain was fixed in 4% paraformaldehyde. The fixed brains were embedded in 1.5% agar blocks containing 5% sucrose and equilibrated in 30% sucrose solution. The frozen blocks were sliced into 16-μm sections using a cryostat microtome and the transverse sectioned slices were mounted on glass slides. The sections were rinsed with PBS several times and enclosed within 75% glycerol by coverslips. Images were obtained from the brain sections of *Tg(gpr151:gal4vp16);Tg(5xuas:spx1:p2a-mCherry)* fish and their siblings. For immunohistochemical analysis, the sections were rinsed with PBS several times and then blocked with 2% bovine serum albumin with sheep serum. After the blocking reaction, the sections were treated with primary antibodies for 2 h at room temperature, washed for 2 h with PBS, and subsequently treated with the appropriate secondary antibodies for 2 h at room temperature. Images were obtained from the sections of the adult brains of *Tg(spx1:mcherry-caax);Tg(pou4f1-hsp70l:gfp)* fish. We used the following antibodies for immunohistochemistry studies: rabbit anti-mCherry/Dsred (1:500, Clontech, Cat. No. 632496) and chicken anti-GFP (1:200, Abcam, Cat. No. AB13970). Alexa Fluor 488 and 647-conjugated secondary antibodies were used for fluorescent detection of primary antibodies (1:1,000, Invitrogen, Cat. No. A-21244 and Abcam, Cat. No. ab96947).

### Novel Tank Test

The novel tank test was performed between 13:00 and 18:00. We followed the protocol that has been described previously (Cachat et al., 2010) with 6–8 months zebrafish. For adaptation, fish were moved to the behavior room 2 h before the test began. Adult behavior analyses were performed using a video camcorder (SONY). Behavior was recorded for 15 min, and the recorded video files were analyzed using the automated tracking software, Ethovision XT 12 (Noldus). We analyzed the behavior for the first 10 min of each recording.

### Total RNA Preparation and Quantitative RT-PCR

The whole-brain or IPN of the adult *Tg(gpr151:gal4vp16);Tg(uas:gfp)* and *Tg(gpr151:gal4vp16);Tg(5xuas:spx1:p*2a-mCherry) zebrafish was isolated and the total RNA was extracted using TRIzol reagent (Invitrogen). Total RNA was extracted from adult brains using TRIzol reagent (Invitrogen). cDNA was synthesized from total RNA using a reverse transcription kit (ImProm-II™ Reverse Transcriptase, Promega). Quantitative RT-PCR was performed using the LightCycler 96 Instrument (Roche). Each reaction mixture contained 2.5 μL of cDNA as a template, 0.2 μM forward and reverse primer, and 2× FastStart Essential DNA Green Master Mix (Roche). PCR was performed under the following conditions: 95°C for 10 min, 40 cycles of 95°C for 10 s, 60°C for 10 s, and 72°C for 10 s. Gene expression of *spexin 1 (spx1), galanin receptor 2a* (*galr2a*), *galanin receptor 2b* (*galr2b*), *plasmacytoma expressed transcript 1 (pet1)*, *tryptophan hydroxylase 2 (tph2)* and *solute carrier family 6*, *member 4a* *(slc6a4a)* was verified using the primers described in Supplementary Table S1.

### Statistical Analysis

All statistical analyses were performed using GraphPad prism 7 software (GraphPad Software). For two-group comparisons, Student’s unpaired *t*-test was used when data were normally distributed, and the Mann-Whitney *U*-test was used when data were not normally distributed. Statistical significance was determined at *p* < 0.05 (two-tailed).

## Results

### Generation of Transgenic Zebrafish Overexpressing *spx1* in the Dorsal Habenula

**Figure 1:**
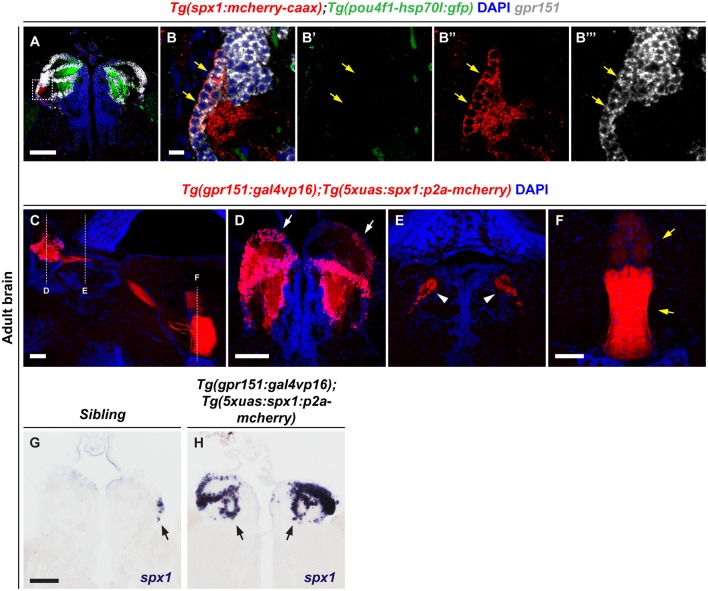
Overexpression of spx1 in the dorsal habenula (dHb). Panels **(A–B”’)** Transverse sections of the habenula of *Tg(spx1:mCherry-CAAX);Tg(pou4f1-hsp70l:gfp)* zebrafish labeled by *gpr151* RNA probe and DAPI. The dorsal aspect is toward the top. Panels **(B–B”’)** are high magnification images of the box area in **(A)**. The yellow arrows indicate the *spx1^+^*/*gpr151*^+^/*pou4f1^−^* cells in the dHb. **(C–F)** Representative images of the adult brain of *Tg(gpr151:gal4vp16);Tg(5xuas:spx1:p2a-mcherry)* zebrafish stained with DAPI. **(C)** Sagittal section view. The dorsal aspect is to the top and the anterior is to the left. Each dotted line marks the transverse section views of every brain region. **(D–F)** Transverse section views. The dorsal aspect is toward the top. **(D)** The habenula. The white arrows indicate mCherry fluorescence in the dHb. **(E)** The fasciculus retroflexus (FR). The white arrowheads indicate mCherry fluorescence in the FR. **(F)** The interpeduncular nucleus (IPN). The yellow arrows indicate mCherry fluorescence in the IPN. **(G,H)** Transverse section views of the habenula in *Tg(gpr151:gal4vp16);Tg(5xuas:spx1:p2a-mcherry)* zebrafish and their wildtype siblings labeled by whole-mount *in situ* RNA hybridization with an *spx1* RNA probe The black arrows indicate *spx1* mRNA expression in the habenula. Scale bar: 100 μm in **(A,C,D–H)** and 10 μm in **(B–B”’)**.

We previously demonstrated that spx1 is expressed in the specific nuclei of dHb, and that dHb SPX1 neurons project to the IPN (Kim et al., 2019). To investigate the role of SPX1 in the dHb, we first confirmed the respective expression patterns of *spx1* and *gpr151*, which are expressed in the dHb (Broms et al., 2015), by fluorescent *in situ* RNA hybridization with *gpr151*, in the brains of* Tg(spx1:mcherry-caax);Tg(pou4f1-hsp70l:gfp)* adult zebrafish. Since *Tg(pou4f1-hsp70l:gfp)* zebrafish express *pou4fl*:GFP in the medial division of dorsal habenula (dHbM; Aizawa et al., 2005), we could distinguish *pou4fl*:GFP^−^ dHb in this transgenic line. We verified that *gpr151* was expressed in the *pou4fl*:GFP^−^ dHb as previously reported (Chou et al., 2016), and found that most of the *spx1*:mCherry^+^ neurons co-express *gpr*151, although *spx1*:mCherry^−^, *gpr*151^+^ cells were also observed (Figures 1A–B). These data indicate that *gpr151*-expressing cells include most of the *spx1*^+^ cells in the dHbL area. To induce overexpression of SPX1 in the dHb, we generated transgenic zebrafish that overexpress SPX1 in the dHb using the GAL4XUAS transactivator system. We produced *Tg(5xuas:spx1:p2a-mCherry)* zebrafish and crossed them with *Tg(gpr151:gal4vp16)* zebrafish that express Gal4vp16 transactivator specifically in the dHb (Chou et al., 2016). *Tg(gpr151:gal4vp16);Tg(5xuas:spx1:p2a-mCherry)* zebrafish grew until adulthood without any morphological defects. To confirm the overexpression of SPX1 in the dHb, we analyzed *spx1*:mCherry fluorescence in the brain of adult *Tg(gpr151:gal4vp16);Tg(5xuas:spx1:p2a-mCherry)* zebrafish. Consistent with our previous observation that dHb SPX1 neurons project into the IPN, mCherry fluorescence was detected in the dHb, fasciculus retroflexus (FR), and IPN in this transgenic zebrafish (Figures 1C–F). Whole-mount *in situ* RNA hybridization with *spx1* in the brain of adult transgenic zebrafish and their wildtype siblings revealed that *spx1* was expressed in the limited area of the dHb of the wildtype siblings, as previously described (Kim et al., 2019), whereas increased *spx1* expression was detected throughout the dHb (Figures 1G,H, Supplementary Figure S1). These data demonstrated that *Tg(gpr151:gal4vp16);Tg(5xuas:spx1:p2a-mCherry)* transgenic zebrafish overexpress *spx1* in the dHb.

### Overexpression of *spx1* in the Dorsal Habenula Reduces Anxiety

**Figure 2:**
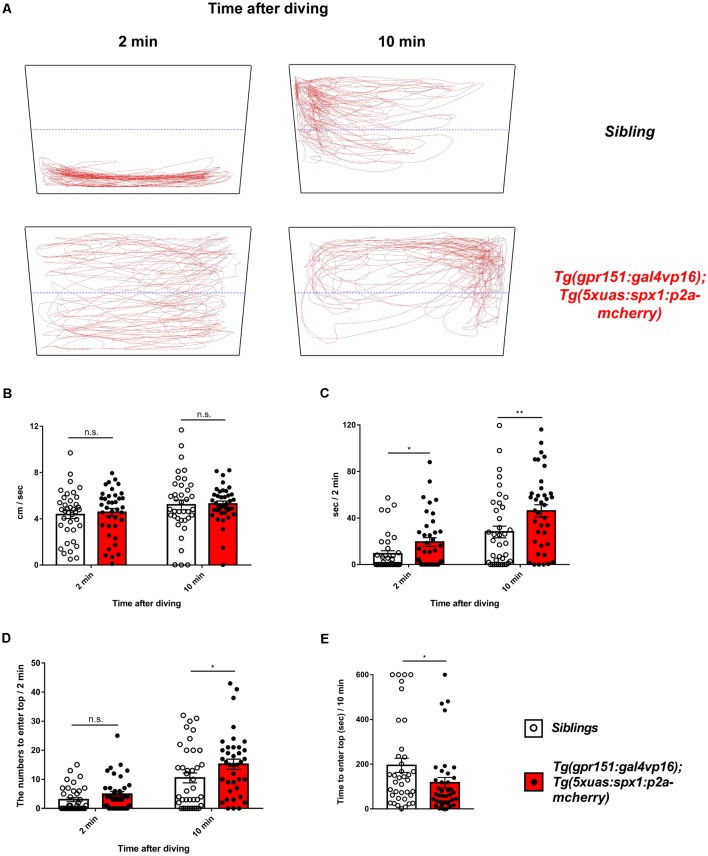
Anxiety behavior of transgenic zebrafish overexpressing *spx1* in the dHb and wildtype siblings. The novel tank test was used to assess the anxiety-related behaviors in adult *Tg(gpr151:gal4vp16);Tg(5xuas:spx1:p2a-mCherry)* zebrafish (dHb-*spx1* OV; *n* = 38) and their wildtype siblings (*n* = 38). **(A)** Representative behavior-tracking results for 2 min at 2 and 10 min after diving. The black quadrangular boxes and blue dotted lines indicate the novel tanks and half lines of tanks. The red lines indicate the tracing lines of the moving fish. **(B–D)** Graph bars and error bars are expressed as mean ± standard error of the mean (SEM). White or black dots in the graphs indicate individual data points. **(B)** The mean velocity at 2 min at 2 and 10 min after diving [2 min: siblings, 4.365 ± 0.3378 cm/s, dHb-*spx1* OV, 4.565 ± 0.3316 cm/s; 10 min (median): siblings, 4.816 cm/s; dHb-*spx1* OV, 5.323 cm/s. n.s., not statistically significant]. The unpaired *t*-test was used for the 2-min analysis and the Mann-Whitney *U*-test for the 10-min analysis. **(C)** Time spent at the top half of the tank for 2 min, at 2 min and 10 min after diving [2 min (median): siblings, 0.02179 s, dHb-*spx1* OV, 11.56 s; 10 min (median): siblings, 19.5 s, dHb-*spx1* OV, 48.88 s; *p < 0.05, **p < 0.005, Mann-Whitney *U*-test]. **(D)** The number of fish that entered the top half of the tank for 2 min, at 2 min and 10 min after diving [2 min (median): siblings, 0.5; dHb-*spx1* OV, 3.5. 10 min (median): siblings, 6.5; dHb-*spx1* OV, 15.5; *p < 0.05, n.s., not significant (Mann-Whitney *U*-test)]. **(E)** The latency to enter the top half of the tank. (Median: siblings, 134.4 s; dHb-*spx1* OV, 65.17 s; *p < 0.05, Mann-Whitney *U*-test).

A previous study has shown that the dHb is associated with anxiety regulation in zebrafish (Mathuru and Jesuthasan, 2013). Thus, we hypothesized that SPX1 might be involved in anxiety regulation in the dHb. To assess this, we performed the novel tank test (Cachat et al., 2010) using the *Tg(gpr151:gal4vp16);Tg(5xuas:spx1:p2a-mCherry)*, which overexpress *spx1* in the dHb, and their wildtype siblings. Interestingly, we observed that transgenic zebrafish overexpressing *spx1* spent more time at the top area of the tank after diving compared with their wildtype siblings (Figures 2A,C). In contrast, both transgenic and wildtype siblings showed similar locomotor activity (Figure 2B). Furthermore, transgenic zebrafish entered the top area of the tank more often at 10 min after diving compared with their siblings (Figures 2A,D). In addition, they have the tendency to visit the top area at 2 min after diving, which is more frequent than that in their siblings; however, this did not reach statistical significance (Figure 2D). Moreover, the latency to enter the top of the tank after diving decreased in the SPX1-overexpressing transgenic fish than wildtype siblings (Figure 2E). Taken together, these results indicated that transgenic fish overexpressing *spx1* show increased anxiolytic behaviors compared with their wildtype siblings, suggesting that SPX1 is involved in the regulation of anxiety in the dHb in zebrafish.

### Anxiolytic Function of SPX1 Is Related With Upregulation of Serotonin-Related Gene Expression

**Figure 3:**
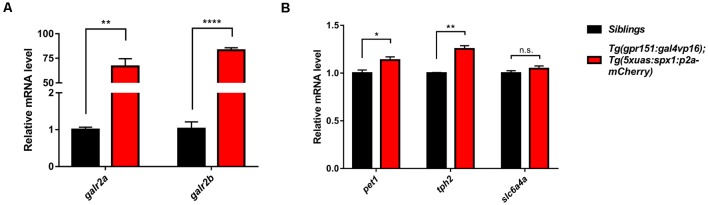
Upregulation of the galanin receptor 2a/2b *(galr2a/b)* and serotonin-related genes in the brain upon overexpression of *spx1* in the dHb. Quantitative RT-PCR in *Tg(gpr151:gal4vp16);Tg(5xuas:spx1:p2a-mcherry)* zebrafish (dHb-*spx1* OV) and their wildtype siblings β-actin expression was used to normalize all samples, and data were acquired in triplicate. Data are represented as mean ± SEM (*n* = 3). **(A)** Relative *galr2a* and *galr2b* mRNA expression in the IPN (*n* = 10 per group): *Galr2a*: siblings, 1.003 ± 0.06386; dHb-*spx1* OV, 66.78 ± 7.778. *Galr2b*: siblings, 1.03 ± 0.1803; dHb-*spx1* OV, 83.17 ± 2.561. **(B)** Relative *plasmacytoma expressed transcript 1* *(pet1)*, *tryptophan hydroxylase 2 (tph2)*, and *slc6a4a* mRNA expression in the brain (*n* = 5 per group): *Pet1*: siblings, 1 ± 0.03215; dHb-*spx1* OV, 1.137 ± 0.03383. *Tph2*: siblings, 1 ± 0.005774; dHb-*spx1* OV, 1.253 ± 0.03333. *Slc6a4a*: siblings, 1 ± 0.02517; dHb-*spx1* OV, 1.047 ± 0.02848 [*p < 0.05, **p < 0.005, ****p < 0.0001 (Unpaired *t*-test), n.s., not significant].

dHb neurons project into the IPN and the IPN is connected to the raphe, which is a part of the serotonergic system (Agetsuma et al., 2010; Okamoto et al., 2012; Chou et al., 2016). Therefore, we postulated that the serotonergic system might be modulated by the SPX1 neuronal pathway from the dHb and involved in the regulation of anxiety. To test this idea, we investigated whether overexpression of *spx1* in dHb increased expression of its putative receptor, *galr2a*, and* galr2b* (Kim et al., 2014). Quantitative RT-PCR with *spx1* and *galr2a/2b*, showed that *spx1* mRNA expression was 10 times higher in the brains of adult *Tg(gpr151:gal4vp16);Tg(5xuas:spx1:p2a-mCherry)* zebrafish compared with their wildtype siblings (Figure 3A). Interestingly, the expression of *galr2a*, not *galr2b*, was significantly increased in the brain of adult *Tg(gpr151:gal4vp16);Tg(5xuas:spx1:p2a-mCherry)* zebrafish compared with their wildtype siblings (Supplementary Figure S2). We further verified the expression of *galr2a* and* galr2b* with quantitative RT-PCR with cDNA isolated from the IPN and discovered that both, *galr2a* and* galr2b* expression was dramatically increased in the adult *Tg(gpr151:gal4vp16);Tg(5xuas:spx1:p2a-mcherry)* zebrafish, when compared with their wildtype siblings (Figure 3A). This suggested that increased SPX1 resulted in an increase in its putative receptor, GALR2a and GALR2b. Next, we examined the mRNA expression of serotonin-related genes in the raphe of adult *Tg(gpr151:gal4vp16);Tg(5xuas:spx1:p2a-mCherry)* zebrafish; most serotonin-related genes were significantly increased in the transgenic zebrafish brains (Figure 3B), such as *plasmacytoma expressed transcript 1* *(pet1)*, *tryptophan hydroxylase 2* *(tph2)* except *solute carrier family 6*, and *member 4a (slc6a4a)*. These data showed that the mRNA expression of the serotonin-related genes was upregulated following the overexpression of *spx1* in the dHb. Taken together, these data suggest that *spx1* overexpression in the dHb partially reduced the anxiety response *via* the modulation of serotonin-related genes in the raphe.

## Discussion

This study is the first to reveal that the function of habenular SPX1 is associated with the regulation of the anxiety response. Overexpression of *spx1* in the dHb reduced anxiety and upregulated *galr2a* and* galr2b* genes in the IPN and serotonin-related genes in the raphe. Although, we could not rule out the possibility of exogenous SPX1 expression in the dHb SPX1-negative neurons, which is caused by the *Tg(gpr151:gal4vp16)* driver line, our results are consistent with the earlier observation that the administration of an SPX-based GALR2-specific agonist induces anti-depressive and anxiolytic effects in mice (Reyes-Alcaraz et al., 2016; Yun et al., 2019), and that Galr2/3 knockout mice expressed an anxiety and depression-like phenotype (Bailey et al., 2007; Lu et al., 2008; Brunner et al., 2014). These data collectively suggest that dHb SPX1 has an anxiolytic function in zebrafish.

Several previous studies have provided indirect evidence suggesting that SPX1 function is implicated in anxiety. First, SPX1 has the capacity to bind Galr2 and Galr3 (Kim et al., 2014), which are implicated in anxiety and depression-like behaviors in mice (Bailey et al., 2007; Lu et al., 2008; Brunner et al., 2014). Second, administration of an SPX-based GALR2-specific agonist induces anti-depressive and anxiolytic effects in mice (Reyes-Alcaraz et al., 2016; Yun et al., 2019). Third, habenula-IPN circuitry is implicated in fear and anxiety responses (Agetsuma et al., 2010; Hikosaka, 2010; Mathuru and Jesuthasan, 2013), and *spx1* is expressed in the specific nuclei of dHb, which projects to the IPN in zebrafish (Kim et al., 2019). However, there is no direct evidence that SPX1 is functionally implicated in anxiety.

Recently, we have shown that SPX1 neurons are located in the dHb and project into the IPN, in which *galr2a* and *galr2b* were expressed, suggesting that dHb SPX1 neurons may interact with GALR2a or GALR2b neurons in the IPN. Further, SPX1-GALR2a/2b neuronal circuits may be involved in the regulation of anxiety (Kim et al., 2019). A recent study has shown that intracerebroventricular administration of SPX1 increased the expression of *Galr2* and *Galr3* mRNA in responses to different types of pain, suggesting that SPX1 stimulates *Galr2* or *Galr3* expression and mediates anti-nociception (Lv et al., 2019). Therefore, we hypothesized that the anxiolytic function of SPX1 in transgenic zebrafish overexpressing SPX1 in the dHb might be mediated by GALR2a or GALR2b in the IPN. Consistent with our hypothesis, we observed increased expression of *galr2a* and *galr2b* upon overexpression of *spx1* in the dHb, suggesting that dHb SPX1 neurons can regulate anxiety *via* interactions with GALR2a and GALR2b in the IPN.

Serotonin-related genes in the raphe were upregulated in transgenic zebrafish overexpressing *spx1* in the dHb. The serotonergic system in the raphe is a major target of the Hb-IPN circuitry in mammals and fish (Herkenham and Nauta, 1979; Okamoto et al., 2012; Chou et al., 2016) and is involved in depression and anxiety (Ressler and Nemeroff, 2000; McDevitt et al., 2011). Previous studies have shown that Galr2 and Galr3 are expressed in the dorsal raphe nucleus (DRN; O’Donnell et al., 1999; Mennicken et al., 2002). In addition, a Galr2 agonist activated the DRN and increased the concentration of serotonin in rats (Mazarati et al., 2005), and Galr3 antagonists attenuated the inhibitory effect on serotonin neurotransmission in mice (Swanson et al., 2005), suggesting that Galr2 and Galr3 in the DRN directly modulate serotonergic neurons. However, our data suggested that SPX1-GALR2a/b mediated Hb-IPN circuits may modulate raphe serotonergic system *via* galr2a/b activation in the IPN. Taken together, our data suggest that SPX1-Galr2a/b in Hb-IPN circuits are implicated in the anxiety response *via* regulation of the serotonergic system in the raphe.

## Data Availability

All datasets generated for this study are included in the manuscript and/or the Supplementary Files.

## Ethics Statement

All experimental procedures were approved by the Korea University Institutional Animal Care and Use Committee (IACUC) and performed in accordance with Animal experiment guidelines of Korea National Veterinary Research and Quarantine Service.

## Author Contributions

IJ, EK and H-CP designed the study, analyzed the data, and wrote the manuscript. EK and IJ performed the experiments. JS advised and contributed to manuscript editing. All authors reviewed the manuscript.

## Conflict of Interest Statement

The authors declare that the research was conducted in the absence of any commercial or financial relationships that could be construed as a potential conflict of interest.
