# Supplementary material for: Overexpression of Spexin 1 in the Dorsal Habenula Reduces Anxiety in Zebrafish
Source: Front Neural Circuits. 2019 Aug 14;13:53. doi: 10.3389/fncir.2019.00053 (PMC6702259; doi:10.3389/fncir.2019.00053)
Supplement: Supplementary file 1 [file Data_Sheet_1.PDF]

Supplementary Material

1.1 Supplementary Table 1. Primer sequences for quantitative RT-PCR

Primer names	Primer sequences
β-actin forward	5’-AGAGCTATGAGCTGCCTGACG-3’
β-actin reverse	5’-CCGCAAGATTCCATACCCA-3’
spx1 forward	5’-CGCACTTGCGCTGTTACTTCTTG-3’
spx1 reverse	5’-GCTGGAGGATGTTCAGGAGAAACG-3’
garl2a forward	5’-GAAACGCGAACTCCACGTAA-3’
garl2a reverse	5’-GAAGGTGCAGATGTCCATGG-3’
garl2b forward	5’-ATGTCTGATCACGAGGACCT-3’
garl2b reverse	5’-CCCAACCTGCCCACTTCTAA-3’
pet1 forward	5’-GCAACTTCGCTCCCATTCA-3’
pet1 reverse	5’-TGGTAGAGGGTTGGAGGAGATC-3’
tph2 forward	5’-TCAGACACTAAAGGAACAACAAAGGA-3’
tph2 reverse	5’-AAGCACAACATTAGGCACAAAGAG-3’
slc6a4a forward	5’-ACATCTCCTCAAAGCCCCAAA-3’
slc6a4a reverse	5’-CCACCAGAGTCCTAAATGTTCCA-3’

Supplementary Figure 1.

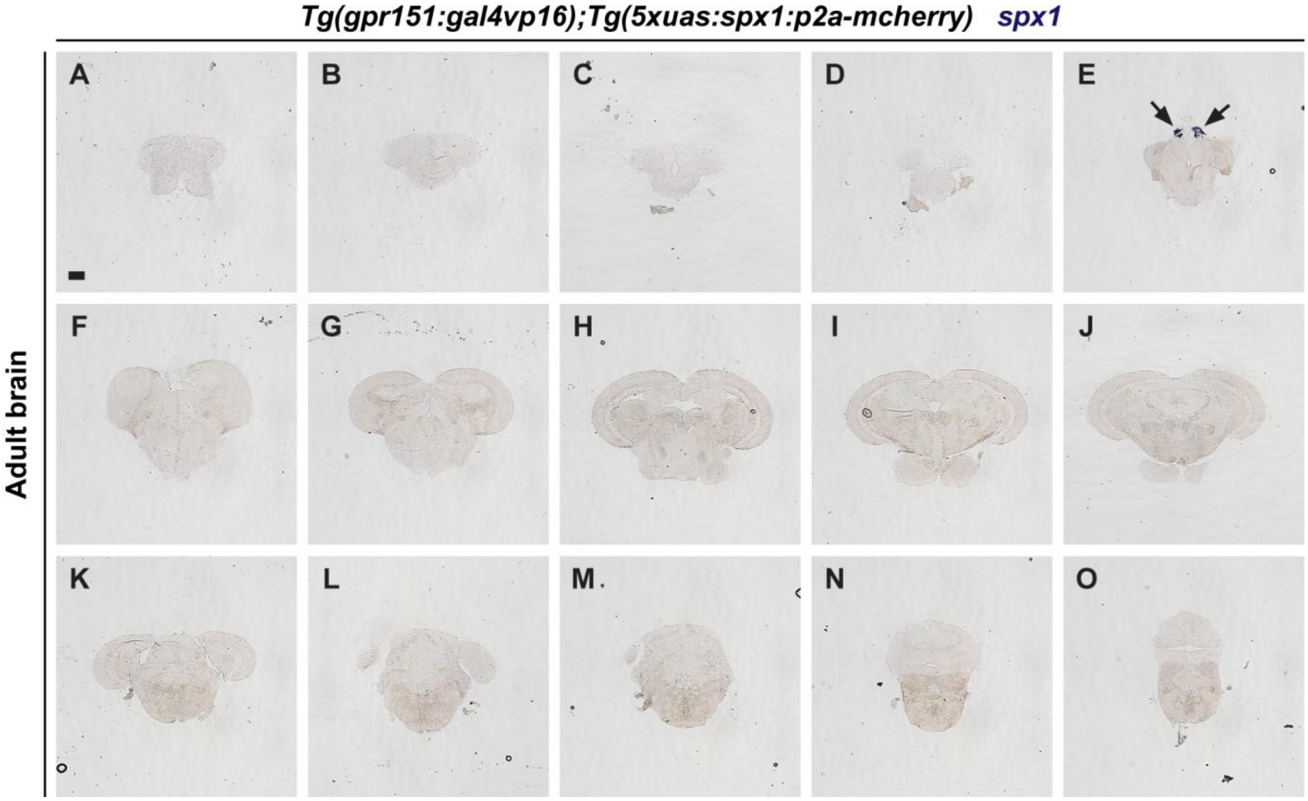

Supplementary Figure 1. Specifically overexpressed *spx1* in the dorsal habenula of the whole adult brain

(A-O) Serial transverse section views of the brain of *Tg(gpr151:gal4vp16);Tg(5xuas:spx1:p2a-mcherry)* zebrafish labeled by *spx1* RNA probe (E) Black arrows indicate overexpressed *spx1* in the dorsal habenula Scale bar: 200 µm

Supplementary Figure 2.

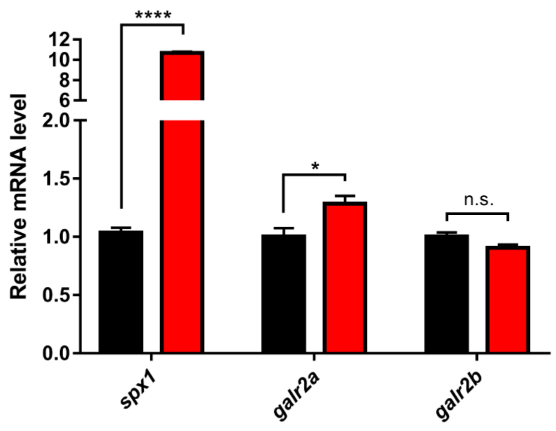

Supplementary Figure 2. Upregulation of *spx1* and *galr2a* in the whole brain upon overexpression of *spx1* in the dHb

Quantitative RT-PCR in *Tg(gpr151:gal4vp16);Tg(5xuas:spx1:p2a-mcherry)* zebrafish (dHb-*spx1* OV) and wildtype siblings (n = 5 per group). β-actin expression was used to normalize all samples, and data were acquired in triplicate. Data are represented as mean ± standard error of the mean (SEM); n = 3. (A) Relative *spx1*, *galr2a,* and *galr2b* mRNA expression: *Spx1*: siblings, 1.037 ± 0.04177; dHb-*spx1* OV, 10.68 ± 0.1323. *Galr2a*: siblings, 1.003 ± 0.07126; dHb-*spx1* OV, 1.283 ± 0.06839. *Galr2b*: siblings, 1.003 ± 0.03528; dHb-*spx1* OV, 0.9067 ± 0.02848. (*$p < 0.05$, ****$p < 0.0001$. Unpaired t-test, n.s.: not significant.)
